# Association between polymorphisms of exon 12 and exon 24 of *JHDM2A* gene and male infertility

**Published:** 2016-06

**Authors:** Zohreh Hojati, Fatemeh Nouri Emamzadeh, Fariba Dehghanian

**Affiliations:** *Division of Genetics, Department of Biology, Faculty of Sciences, University of Isfahan, Isfahan, Iran.*

**Keywords:** *Histone Demethylases*, *Infertility*, *Polymerase Chain Reaction*

## Abstract

**Background:**

Some dynamic changes occurs during spermatogenesis such as histone removal and its replacement with transition nuclear protein and protamine. These proteins are required for packing and condensation of sperm chromatin. JHDM2A is a histone demethylase that directly binds to promoter regions of *Tnp1* and *Prm1* genes and controls their expression by removing H3K9 at their promoters.

**Objective:**

The association between polymorphisms of exon 12 and exon 24 in *JHDM2A *gene and male infertility were evaluated for the first time.

**Materials and Methods:**

In this experimental study, 400 infertile men (oligospermia and azoospermia) and normal healthy fathers were evaluated (n=200). Single Strand Conformation Polymorphism (SSCP-PCR) and polymerase chain reaction-restriction fragment length polymorphism (PCR-RFLP) methods were used for screening any polymorphisms that are exist in exon 12 and exon 24.

**Results:**

Exon 24 PCR products were analyzed by RFLP but no polymorphism was found in this exon at the restriction site of *Eco*RV enzyme. Our monitoring along the whole nucleotides of exon 12 and exon 24 were continued using SSCP method, but we found no change along these exons.

**Conclusion:**

Generally, this study evaluated the association between polymorphisms in exon 12 and exon 24 of *JHDM2A *gene and male infertility which suggests that polymorphisms of these exons may not be associated with the risk of male infertility.

## Introduction

Infertility is a complex disorder which affects both men and women within reproductive age group. Many abnormalities and genetics alterations are involved in infertility physiology and etiology. Recent studies indicate a critical role of epigenetic regulatory mechanisms relating to male infertility (1). Epigenetic mechanisms including DNA methylation, noncoding RNAs and modifications of histone proteins may regulate the genes expression level which are involved in infertility (2).

Histone modifications are defined as important post-translational regulatory codes which are essential for appropriate gene function. Methylation, phosphorylation, acetylation, sumoylation, and ubiquitylation are some of the most important modifications which affect the amino acid of N-termini histone tails (3). Methylation of Histone3 Lysine9 (H3K9) is a type of classic histone modifications which commonly results in gene silencing at heterochromatin regions (4). H3K9 methylation has important roles during mammalian spermatogenesis and is precisely regulated at multiple spermatogenesis stages. jmjC-containing histone demethylase 2a (JHDM2A) is a H3K9 demethylase and it is also known as JMJD1A or KDM3A. The gene encoding *JHDM2A* is located on human chromosome 2p11.2 and contains 26 exons with 51486 base pairs length (5). JHDM2A has critical regulatory functions relating the spermatogenesis control and metabolism. The expression level of fat metabolic genes in muscle and brown fat tissues is regulated by JHDM2A (6). During spermatogenesis, the expression level of JHDM2A increases up to 70 folds and its highest level of expression was detected in round spermatids. JHDM2A binds to promoter region of transition nuclear proteins 1 (TNP1) and protamine-1 in order to increase their expression through demethylation of H3K9 modification (7, 8). 

DNA in mature sperms should be enough packed to set in a small and compact sperm nucleus. The expression of post meiotic proteins such as TNP and protamines is essential for replacing with histone and formation of weaved DNA. In fact, the assembly of sperm nucleus is necessary for conversion of round spermatids to mature sperms and defection in chromatin condensation results in production of impaired sperms. JHDM2A is essential for spermiogenesis as it directly controls the expression of post meiotic proteins requiring for sperm nucleus compaction. The JHDM2A deficiency is related to male infertility because immature sperms can't pass through the ovule membrane and can't penetrate an egg successfully (9). 

In this study, we examined the association between polymorphisms in exon 12 and exon 24 of *JHDM2A *gene and male infertility. Exon 12 is prone to a frame shift (rs35743643) at position of amino acid 632 by insertion of adenine which causes a shift in reading frame and alters the gene product. This frame shift occurred before jmjC and zinc finger domains and resulted in production of impaired protein*.* Our in silico analyses indicate that this frame shift has main role in alteration of JHDM2A activity due to its effects on jmjC and zinc finger domains which are necessary for enzyme activity. Exon 24 is also prone to nonsense polymorphism at position of amino acid 1193 (rs17853822) that causes a premature stop codon in the transcribed mRNA and formation of truncated, non-functional protein. These polymorphisms could be a risk factor in susceptibility of male infertility. 

In this work, the existence of any polymorphism in exon 12 and exon 24 of *JHDM2A *gene was investigated in human for first time. So the results could expand our knowledge about the association between this gene and human male infertility.

## Materials and methods

**Subjects**

In this case-control study 200 healthy volunteer fathers who referred to clinic for a checkup and 200 infertile men who attend the Isfahan Fertility-Infertility Center and Royan Institute during the period of April to July 2013 were analyzed. Semen and blood sampling was performed according to the patient consent and an agreement between the University of Isfahan and Isfahan Fertility- Infertility Center and Royan Institute. 

Blood samples were collected in EDTA and heparin coated tubes and were stored at-20^o^C for further analyses. Semen samples were analyzed according to WHO criteria. A few factors are normally determined during Semen analysis, such as volume, sperm count, sperm motility and sperm morphology. According to the data obtained from the semen analysis, these men were categorized in two groups including oligospermia and azoospermia.

**DNA genotyping**

Genomic DNA was extracted from leukocytes of peripheral blood samples using Miller salting-out method (10). The quantification of genomic DNA was then determined using agarose gel electrophoresis (1%) and spectrophotometer. All genomic DNAs were diluted in 0.5 M TE and stored at -20^o^C for further genotyping analyses. To analyze polymorphisms in exon 12 and exon 24 of *JHDM2A *gene, PCR-RFLP SSCP methods were performed.

**PCR reactions**

Two sets of primers were designed by Oligo ®7 in order to analyze polymorphisms of exon 12 and exon 24 (Table I). PCR reactions were performed in 25 µl reaction mixture containing 1 µl of template DNA (100 ng/µl), 2.5µl of 10× PCR Buffer (20 mM Tris-HCl pH 8.6, 50 mM KCl, Cinnagen Inc, Iran), 1 µl of MgCl_2_ (50 mM, Cinnagen Inc, Iran), 0.5 µl of each forward and reverse primer (10 pM/µl), 0.5 µl of dNTPs mix (10 mM, Cinnagen Inc, Iran), 0.3 µl of Taq DNA polymerase (Cinnagene, Co., Iran). The PCR program was as follow for exon 12: primary denaturation 94^o^C 4 min, 30 cycles of denaturation 94^o^C 45 sec, annealing 62^o^C 45 sec, extension 72^o^C 1 min, and an extra extension 72^o^C 10 min. 

The PCR program was also adjusted for exon 24 as following order: primary denaturation 94^o^C 4 min, 30 cycles of denaturation 94^o^C 1 min, annealing 50^o^C 30 sec, extension 72^o^C 30 sec, and a final extra extension 72^o^C 10 min. Each PCR products was run on 1% agarose gel and was visualized using ethidium bromide staining.

**SSCP and RFLP analyses**

Polyacrylamide gel electrophoresis was used for PCR products analyzing which are related to exon 12 and exon 24. 12% non-denaturing polyacrylamide gel was used for SSCP with 5% glycerol and 5% TBE buffer. Each 50 ml solution contains; glycerol 5 ml, TBE 10×5 ml, acrylamide 40% 15 ml, ammonium persulfate 0.1 gr/L 1 ml, ddH_2_O 24 ml, TEMED 30 µl. SSCP buffer contains; formamide 95%, NaOH 10 mM, Bromophenol Blue 0.025%, Xylen Cyanol 0.025%. PCR products were mixed with SSCP buffer and then denatured at 95^o^C for 5 min and rapidly cooled on ice. Samples were loaded on polyacrylamide gel and SSCP processes were performed at 250 V for 20 hrs. 

Finally, silver staining method was done for gel staining. At this point, each single strand DNA forms special conformation based on its sequence, so by using polyacrylamide gel electrophoresis polymorphisms are recognizable. Observation of two bands means that there was no polymorphism because one of these bands was related to the 5'-3' DNA strand and the other one was related to the 3'-5' DNA strand. In case of polymorphism, two bands in different position compare to normal bands or even more than two bands may be detected.

In order to determine the nonsense polymorphism of exon 24 at position of amino acid 1193 (SerThr) RFLP analysis was also performed. Restriction analysis was done using 4 units of *Eco*RV enzyme (Fermentas, Vilnius, Lithuania) in *Eco*RV buffer solutions (10×, Fermentas) at 37^o^C for 1 hr. The restriction fragments were analyzed on 1.5% agarose gel.

**Statistical analysis**

Statistical analysis was done using SPSS version 16 software (SPSS, Inc., Chicago, IL, USA). The Chi-square distribution (χ^2^) was used to evaluate the association between polymorphisms of exon 12 and exon 24 of *JHDM2A* gene and male infertility.

## Results

**Distribution of exon 12 polymorphism**

**Figure 1:**
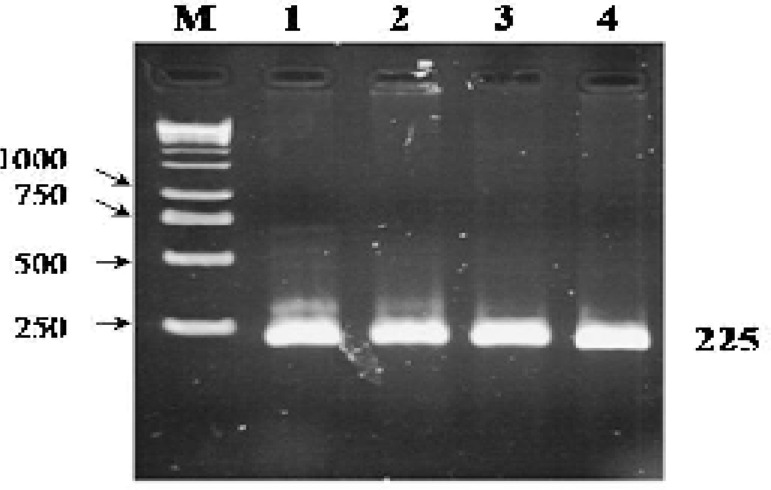
Amplification of exon 12 using specific primers. A fragment of exon 12 (225 bp) was successfully amplified through PCR reaction. 1 Kb DNA Marker (lane M). A 1% agarose gel was used here for gel electrophoresis. All of the numbers are in bp

**Figure 2:**
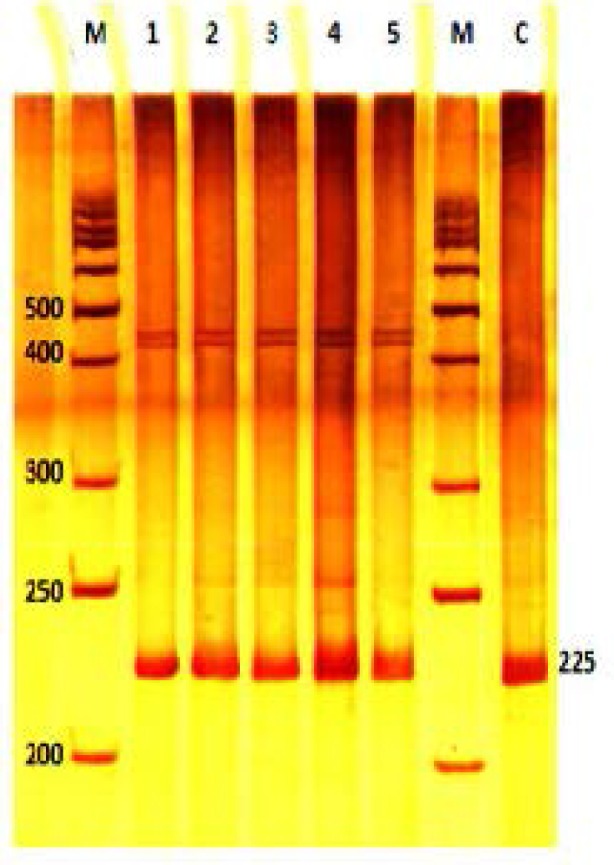
Polyacrylamide gel of exon 12 after SSCP analysis. Samples 1 and 2 are controls, samples 3, 4 and 5 are infertile samples. Single strand bands in both fertile and infertile samples are in the same position and no polymorphism is detected. 50 bp DNA Marker (lane M), a 225 fragment which isn’t treated with SSCP buffer (lane C

Using suitable forward and reverse primers which were designed for exon 12 of *JHDM2A* gene, a PCR product (225 bp) was amplified. The quality and quantity of PCR products were examined by 1% gel agarose (Figure 1). Using SSCP method for exon 12 PCR product that followed by polyacrylamaide gel electrophoresis, only two bands was detected for both fertile and infertile samples in the same position which means that there was no polymorphism (Figure 2).

**Analysis of exon 24 polymorphism using RFLP and SSCP analysis**

**Figure 3:**
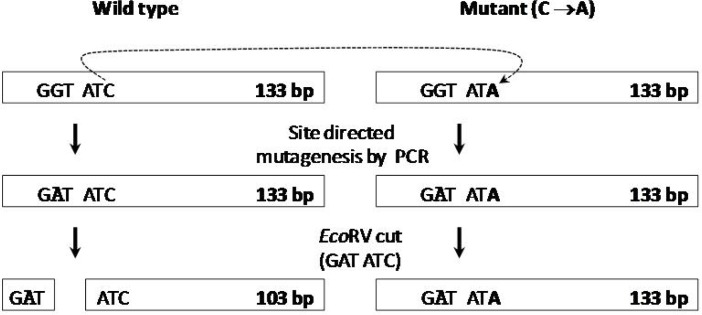
Schematic illustration of *Eco*RV recognition site for normal and mutant samples. The recognition site of *Eco*RV is detected in normal samples without polymorphism and it isn’t detected in samples with polymorphism. As clearly shown, a site directed mutagenesis was performed to form *Eco*RV recognition site using specific primers

 It was found that nucleotide C which is prone to nonsense polymorphism with its neighbors can be recognition site for *Eco*RV by the replacement of G with A in position of amino acid 1192 in normal genome (Figure 3). In mutant samples, this replacement makes no difference. It means that after amplification there will be *Eco*RV recognition site for wild type PCR products, but not for mutant PCR products. 

**Figure 4:**
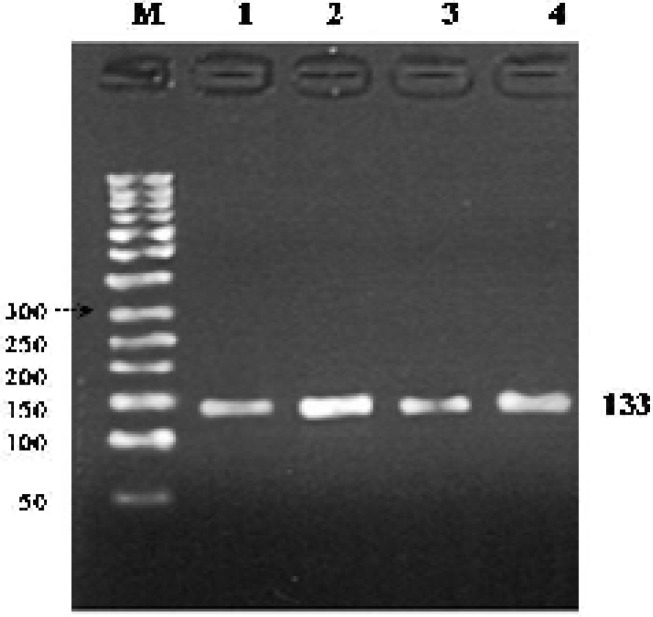
PCR product of exon 24. A fragment of exon 24 (133 bp) was successfully amplified through PCR reaction. 50 bp DNA Marker (lane M). A 1% agarose gel was used here for gel electrophoresis. All of the numbers are in bp

**Figure 5:**
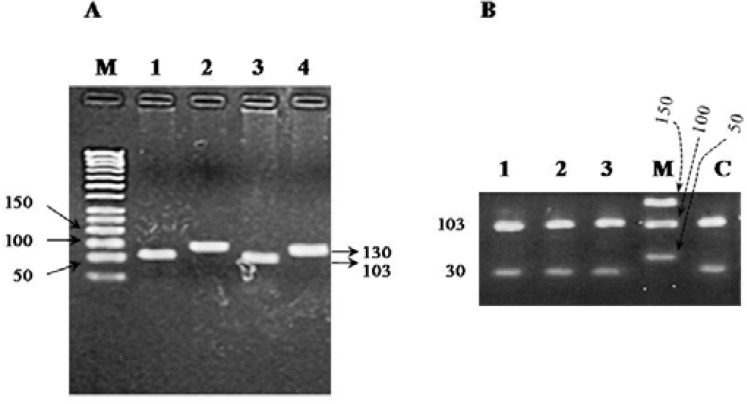
Detection of C A polymorphism in exon 24 of *JHDM2A *gene. After treatment of PCR product with *Eco*RV enzyme, both control and infertile samples showed two fragments. It reveals that no sample has polymorphism at predicted site. A) A 1.5% agarose gel was used here for gel electrophoresis. All of the numbers are in bp. 50 bp DNA Marker (lane M), digested PCR product of fertile sample (lane 1), un-digested PCR product of fertile sample (lane 2), digested PCR product of infertile sample (lane 3), un-digested PCR product of infertile sample (lane 4). B) For detecting the 30 bp band, ethidium bromide staining was performed for more 30 min

**Figure 6:**
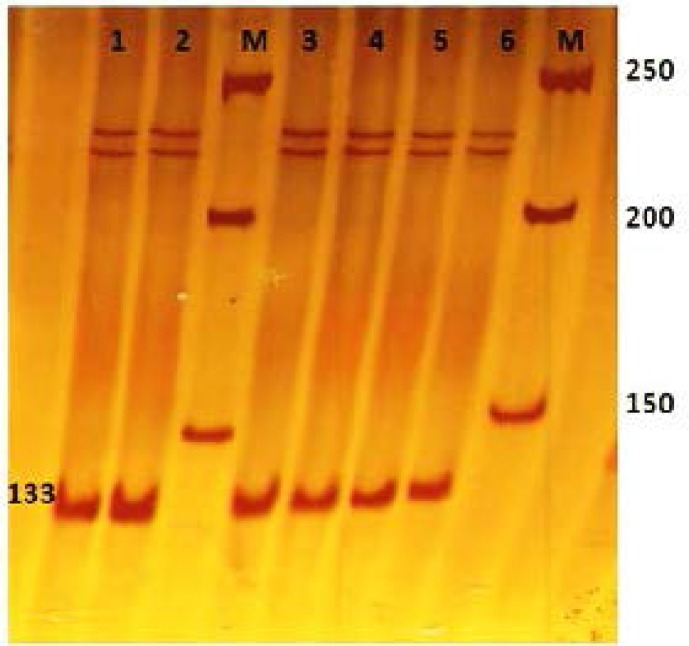
Polyacrylamide gel after applying SSCP technique for exon 24 PCR products. All single strand bands are in the same position and no polymorphism is detected. 50 bp DNA Marker (lane M), fertile samples (lane 1, 2), infertile samples (lane 3, 4, 5, 6

Therefore a set of suitable primers were designed for this exon. Forward primer had A instead of C at situation of nucleotide 20 and can cause a polymorphism at this point during PCR. After amplification integrity of PCR products were tested by 1% agarose gel (Figure 4). Gel electrophoresis indicates that both fertile and infertile samples showed two bands and this polymorphism didn't detect between these samples (Figure 5). SSCP was also performed on PCR products to search for whole exon 24, but only two bands at same position were detected for both fertile and infertile samples. Therefore no polymorphism has been detected in this region (Figure 6).

**Table I:** Two set primers that were designed for screening of polymorphisms in exon 12 and exon 24 of *JHDM2A *gene

**Exon**	**Name**	**Primer sequence**	**PCR product**
12	F12	5'- GCTTCCCCTAGGTTACAATTCAACAAACAT-3'	225 bp
	R12	5'-GTGTGGCTCAATAGTTGACATAGCTTCCTT-3'	
24	F24	5'-GTTGCATAATATTTTGAATGTATTCTAGAT-3'	133 bp
	R24	5'-ATACTCTTGATGAAGACGTTTTCTTAATGA-3'	

## Discussion

Dynamic genetic and epigenetic alterations during mammalian spermatogenesis are defined as important biological processes. Several regulatory processes in testes and male germ cells result in a distinct epigenetic pattern for this tissue compare to other tissues. It has been clarified that structural changes of chromatin which are caused by histone modifications plays a vital role in germ cell development. During spermatogenesis, round spermatids undertake many changes to become a mature sperm, including formation of tight condensed DNA in a tiny nucleus (1, 2). 

DNA in mature sperms should be enough packed to set in a small and compact sperm nucleus, and it needs the expression of vast groups of post meiotic proteins such as TNP and protamines to be replaced with histone (11). DNA becomes twisted into packed loops form through this replacement. Moreover, post meiotic chromatin condensation defect was seen in Jhdm2a- deficient mice. JHDM2A is defined as an enzyme which involves in chromatin condensation. It specifically expresses in male germ cells at meiotic and post meiotic stages (9). JHDM2A is a specific demethylase for mono and dimethylated H3K9 residues with preference for dimethylated residues. JHDM2A is about 147.2 KD and contains 1321 amino acids (12). 

JHDM2A is formed of a couple of domains, including jmjC and a zinc finger domain which are both involved in demethylation activity (13). jmjC domain is formed of an alpha helixes domain and eight beta sheets in form of a dynamic enzymatic pocket and is necessary for JHDM2 demethylation activity. Fe (II) and alpha-ketoglutarate are enzyme cofactors. jmjC demethylates H3K9 in an oxidative reaction that generates formaldehyde and succinate. It seems that this demethylation occurs by direct hydroxylation of methyl group and generation of unstable product containing a hydroxy-methyl release spontaneously in form of aldehyde (14). 

JHDM2A binds to the promoter region of TNP1 and protamine-1 and by demethylation of the H3K9 residues at these regions, causes an increase in expression of protamines and TNP1 requiring for condensation of sperm chromatin (15). So JHDM2A is essential for spermiogenesis as it directly controls the expression of post meiotic proteins requiring for sperm nucleus compaction (16). The JHDM2A deficiency is related to male infertility and inhibition of JHDM2A expression by siRNA affects on expression of JHDM2A-related genes (17, 18). 

Generally, 75% of male infertility are described as idiopathic and there isn’t any well-known molecular mechanism relating to these defects (19). So, many researchers focus on finding the new molecular processes which are critical in male infertility in order to develop more efficient diagnosis and therapeutic methods. Originally, *JHDM2A *gene was reported as a testis-specific gene transcript by Okada *et al* (20). They indicated that *JHDM2A* gene plays an important role in transcriptional activation of some testis specific genes. In fact, the co-expression of this gene with RNA polymerase II shows that *JHDM2A* gene could be important for transcriptional activation of some testis specific genes. The infertility and smaller testes were also reported in JHDM2A-deficient mice (21). 

In 2010, Okada *et al* determined some important roles for JHDM2A in spermatogenesis and regulation of fat metabolic in muscle and brown fat tissue (5, 22). Recently, Najafipour *et al *evaluated the expression level of *YBX2* and *JHDM2A* genes in testicular tissues of men with non-obstructive azoospermia for the first time. Their results specified that *YBX2* and *JHDM2A *genes may have an important role in male infertility and these two genes can be helpful biomarkers for diagnosis of male infertility (23). The protein levels and mRNA expression of *JHDM2A* as an epigenetic-related gene were also evaluated in fresh and cryopreserved boar spermatozoa using ELISA and qRT-PCR. *JHDM2A* mRNA expression and protein level were decreased after cryopreservation or freezing in comparison with fresh spermatozoa (24, 25). 

According to these results, we evaluated the polymorphisms of exon 12 and exon 24 of *JHDM2A *gene. Two residues of exon 12 and exon 24 are prone to frame shift and nonsense polymorphism respectively. According to the in silico analyses, these two polymorphisms result in production of truncated or inefficient JHDM2A protein*. *Despite the fact that many studies showed the important role of this gene in male infertility, our results did not show significant association between polymorphisms of exon 12, exopn 24 and male infertility. On the other hand, O'Bryan *et al* indicates that although the disruption of several mice genes leads to male infertility, but very few human patients with that mutated genes show male infertility (26). 

Altogether, the role of polymorphisms in exon 12 and exopn 24 of *JHDM2A *gene is not very significant in male fertility; therefore, the polymorphism of other exons and more samples must be investigated. In addition, more comprehensive studies in larger Iranian population or in other populations are needed to establish the association of these polymorphisms with male infertility. It is hoped that the results of this study will provide a basis for further researches to explain the possible roles of *JHDM2A *gene and its polymorphisms in male infertility.

## Note

***This article extracted from M.Sc. thesis. (Fatemeh Nouri Emamzadeh)***
